# Metabolite profiling and molecular dynamics-based *in silico* evaluation of methanolic leaf extracts of *Stevia rebaudiana* Bertoni against α-amylase and DPP-IV

**DOI:** 10.3389/fpls.2026.1859134

**Published:** 2026-09-28

**Authors:** Aswin Sakthivel Manoharan, Ramesh Kumar Selvan

**Affiliations:** VIT School of Agricultural Innovations and Advanced Learning (VAIAL), Vellore Institute of Technology, Vellore, Tamil Nadu, India

**Keywords:** antidiabetic, dipeptidyl peptidase IV, *in silico* study, molecular docking, molecular docking simulation, α-amylase

## Abstract

*Stevia rebaudiana* Bertoni is a natural sweetener with reported antidiabetic properties; however, the molecular basis underlying the interactions of its major phytochemicals with key glycaemic-regulating enzymes remains insufficiently understood. The present study employed an integrated *in silico* workflow to evaluate the antidiabetic potential of selected steviol-derived phytochemicals against human pancreatic α-amylase and dipeptidyl peptidase-IV (DPP-IV). Methanolic leaf extracts were profiled by GC–MS, and steviol, together with the principal steviol glycosides (stevioside, rebaudioside A, and rebaudioside D), was selected for computational analyses. Pharmacokinetic properties were predicted using SwissADME, followed by molecular docking and 100 ns molecular dynamics simulations incorporating principal component analysis (PCA), free-energy landscape (FEL), dynamic cross-correlation analysis (DCCM), and hydrogen-bond analysis, together with MM-PBSA binding free energy and per-residue energy decomposition. Among the evaluated phytochemicals, steviol exhibited the most favourable predicted docking profile against α-amylase (−8.46 kcal/mol), whereas rebaudioside A showed the most favourable predicted docking profile against DPP-IV (−8.34 kcal/mol). Molecular dynamics and complementary structural analyses indicated stable conformational behaviour and provided additional insights into the dynamic and energetic characteristics of the selected protein–ligand complexes. ADME analysis further demonstrated distinct pharmacokinetic characteristics among the evaluated phytochemicals, with steviol exhibiting comparatively more favourable predicted oral drug-likeness, whereas the steviol glycosides displayed physicochemical properties that may favour gut-localised α-amylase inhibition. These findings provide computational evidence supporting the potential interactions of steviol-derived phytochemicals with α-amylase and DPP-IV. However, the results are based entirely on computational predictions and require experimental validation through *in vitro* enzyme inhibition assays, pharmacokinetic studies, and *in vivo* investigations before their therapeutic potential can be established.

## 1 Introduction

Stevia, commonly known as Honey leaf, is a natural sweetener made from the *Stevia rebaudiana* Bertoni plant, which is a member of the Asteraceae family, referred to as the “sunflower family” (Sadhu et al., 2024). The leaves of Stevia have been traditionally used as a natural sweetener and medicinal plant for several centuries by indigenous communities in South America (Hanna et al., 2023). The sweetness of the Stevia plant was discovered by the Italian-Swiss botanist Dr Moisés S. Bertoni in 1901. The sweetness of the stevia plant is given by steviol glycosides, a group of sweet compounds that are synthesised in the leaves (Thakur et al., 2025). Stevia is consumed as a natural sweetener and also contains numerous bioactive effects, such as antifungal, anticancer, antioxidant and immune system-enhancing modulators (Medeot et al., 2023; M. and S., 2025). Steviol glycosides found in *Stevia rebaudiana* leaves have demonstrated considerable potential for the management of Type 2 Diabetes Mellitus (T2DM). These glycosides, in particular, stevioside and rebaudioside A, enhance insulin secretion and insulin sensitivity, thereby contributing to improved glycaemic regulation (Anker et al., 2019). The health-promoting nature of foods is attributed to their nutrient composition and the bioactive phytochemicals (Mishra et al., 2024). Stevia has been reported to suppress hepatic glucose production and reduce intestinal glucose absorption. The development of Stevia as a natural adjunct in the management of Type 2 Diabetes Mellitus is promising as it has been reported to reduce fasting blood glucose and HbA1c levels (Ahmad et al., 2024). In a 16-week randomized, placebo-controlled trial in patients with type 2 diabetes, daily consumption of 1000 mg rebaudioside A did not significantly affect HbA1c, fasting glucose, insulin, C-peptide, blood pressure, body weight, or lipid parameters compared with placebo. Rebaudioside A was well tolerated and was not associated with an increased incidence of hypoglycemia (Maki et al., 2008). A 90-day pilot study in 45 overweight participants found that replacing added sugar with a stevia-based sweetener was associated with significant reductions in body weight and waist circumference, while HbA1c showed no significant change. No adverse outcomes were reported during the study period (Raghavan et al., 2023).

Diabetes is a chronic disorder that starts as a result of insulin insufficiency in the pancreas or the body cannot effectively utilise insulin, which is a hormone that controls the level of blood glucose (Hua, 2020). Approximately 830 million people around the world suffer from diabetes mellitus (DM), and hence it is considered a serious public health concern. The majority of this population lives in countries with low or middle incomes (Essaouiba et al., 2022). More than half of diabetics do not receive treatment for their disease. Insulin resistance or insufficient insulin secretion is directly linked to the pathogenesis of diabetes mellitus (DM), which results in excessive blood glucose levels (Teja et al., 2020). Uncontrolled diabetes mellitus can lead to serious problems, such as cardiovascular and kidney disease, that may significantly increase medical expenses (Kharroubi, 2015; Wu et al., 2024). The increasing prevalence of childhood obesity is the main reason for the development of type 2 diabetes in teenagers and young adults during their adulthood (Gayathiri et al., 2025).

The enzyme α-amylase facilitates the hydrolysis of α-1,4-glucan linkages found in starch, maltodextrins, and similar carbohydrates, playing a vital role in the decomposition of polymeric substances (Fadimu et al., 2022). Significant amounts of maltose are produced as a result of the dietary starch and carbohydrate digestion, which is carried out by the enzyme α-amylase (Khadayat et al., 2020). Then, α-glucosidase breaks down the maltose to produce glucose, which the human gut may absorb (Ahmad et al., 2017a). α-glucosidase enzymes are located on the brush border of intestinal mucosal cells in humans. When the α-1,4 glycosidic bond is hydrolysed, breaking down the glucose units from the non-reducing end of the polysaccharide chain (Ahmad et al., 2021).

The prolonged use of synthetic drugs has been associated with adverse side effects, including hypoglycemia, hepatotoxicity, flatulence, diarrhoea, and stomach pain (Van De Laar et al., 2005). Hypoglycaemic medications such as metformin, acarbose, voglibose and miglitol aid in regulating blood glucose levels, but some may be associated with adverse gastrointestinal side effects (Picot et al., 2014). The utilisation of traditional medical systems to treat economically expensive diseases like diabetes is currently gaining popularity in developing countries. This is because herbal-based drugs are affordable, effective, although their safety and possible adverse effects require appropriate evaluation (Mogole et al., 2020). Hence, plant-based therapies can be revitalised and utilised as a safer alternative for diabetes treatment (Lin et al., 2016).

Computational approaches have become indispensable tools in natural product-based drug discovery by enabling the rapid identification and characterization of bioactive compounds and their interactions with therapeutic targets. Among these, GC–MS facilitates metabolite profiling, while ADME prediction provides insights into pharmacokinetic behaviour and drug-likeness. Molecular docking predicts the binding mode and estimates the binding affinity between phytochemicals and target proteins, whereas molecular dynamics simulations further evaluate the conformational stability and dynamic behaviour of protein–ligand complexes under physiological conditions (Bikadi and Hazai, 2009; Utomo and Rifa, 2012). Collectively, these complementary approaches provide a comprehensive framework for elucidating the molecular basis of bioactivity and prioritising promising lead compounds. However, computational predictions should be interpreted as supportive evidence and require subsequent experimental validation through *in vitro* and *in vivo* studies.

*Stevia rebaudiana* has well-documented antidiabetic potential, but the molecular mechanisms underlying its activity against two key glycaemic-regulating enzymes (α-amylase and DPP-IV) remain incompletely understood. Most previous computational studies focused on individual steviol glycosides or a single molecular target, whereas the current study evaluates multiple metabolites identified from the methanolic leaf extract. Based on the reported antidiabetic properties of *Stevia rebaudiana*, we hypothesized that metabolites identified from its methanolic leaf extract, particularly steviol glycosides and related phytoconstituents, possess favourable molecular interaction potential and pharmacokinetic characteristics suitable for further investigation and can effectively interact with the therapeutic targets α-amylase and DPP-IV, thereby providing a molecular basis for the plant’s antidiabetic activity. The present study provides an integrated evaluation of GC–MS-identified Stevia metabolites against two complementary diabetes-related targets, combined with ADME profiling, molecular docking, 100 ns molecular dynamics simulations, PCA, free-energy landscape analysis, dynamic cross-correlation analysis, hydrogen-bond analysis, and per-residue MM-PBSA decomposition. This integrated approach provides additional structural and energetic insight into the predicted interactions of steviol-derived phytochemicals with α-amylase and DPP-IV.

Among the therapeutic targets associated with type 2 diabetes mellitus, α-amylase and dipeptidyl peptidase-IV (DPP-IV) play complementary roles in maintaining glucose homeostasis. α-Amylase initiates the hydrolysis of dietary starch into absorbable carbohydrates, and its inhibition delays glucose absorption, thereby reducing postprandial hyperglycaemia. In contrast, DPP-IV rapidly degrades incretin hormones such as GLP-1 and GIP, which are responsible for glucose-dependent insulin secretion. Inhibition of DPP-IV prolongs incretin activity, enhances insulin secretion, and improves glycaemic control. Steviol glycosides, particularly stevioside and rebaudioside A, have been reported to improve glucose homeostasis through multiple mechanisms, including enhanced insulin secretion, improved insulin sensitivity, antioxidant activity, and modulation of carbohydrate metabolism. These multifunctional properties suggest that steviol glycosides and related phytoconstituents may interact with key enzymes involved in glucose regulation, such as α-amylase and DPP-IV, thereby providing a mechanistic basis for their reported antidiabetic effects (Kim et al., 2018; Sahoo et al., 2024; Thottappillil et al., 2024).

## 2 Materials and methods

### 2.1 Sample preparation and GC-MS analysis

The stevia plants have been obtained from Gandhi Krishi Vigyana Kendra (GKVK), University of Agricultural Sciences, Bangalore, India and are grown under shade net conditions in the Nursery, Vellore Institute of Technology, Vellore, Tamil Nadu, India. Fresh leaf samples weighing 100 grams were collected from the plants, dried and then used to prepare a methanolic extract of stevia leaves. The methanolic leaf extract of dried Stevia leaves was analysed using GC-MS. Steviol (hydroxydehydrostevic acid), identified by GC–MS analysis, together with stevioside, rebaudioside A, and rebaudioside D, which are the principal steviol glycosides of *Stevia rebaudiana* reported in previous phytochemical studies (Bergs et al., 2012; Aranda-González et al., 2014, 2015), were selected for subsequent computational analyses because of their well-documented antidiabetic potential. The selected ligands are presented in **Table 1**. These compounds exhibit a variety of biological properties, including anticancer, antidiabetic, antimicrobial, antioxidant, and anti-inflammatory action (Kolb et al., 2001; Wang et al., 2015; Hanna et al., 2023).

**Table 1:** Selected ligands for molecular docking.

S.NO	Category	Pub ID	Selected ligand
1	VOLATILE	6918840	Rebaudioside A
2	VOLATILE	71773169	Rebaudioside D
3	NON-VOLATILE	452967	Steviol (Hydroxydehydrostevic acid)
4	VOLATILE	442089	Stevioside

GC–MS analysis was performed using an Agilent 5975C GC–MS system equipped with a DB-5MS column (30 m × 0.25 mm I.D. × 0.25 µm film thickness), with helium as the carrier gas at a flow rate of 1.0 mL/min. A 1 µL sample was injected in split mode (1:5) at an injector temperature of 250 °C. The oven temperature was initially set at 50 °C for 1 min, increased at 15 °C/min to 250 °C and held for 5 min, followed by an increase at 20 °C/min to 310 °C and held for 16 min, with a post-run temperature of 50 °C. Compounds were ionized by electron ionization (EI) at 70 eV, and mass spectra were acquired over m/z 50–350 with ion-source and transfer-line temperatures of 230 °C and 280 °C, respectively. Data were processed using MassHunter, and compound identification was performed by comparison with spectra in the NIST mass spectral library. A limitation of GC–MS is that it primarily provides qualitative or semi-quantitative information based on chromatographic retention and mass-spectral matching; therefore, library-based identification alone does not provide definitive structural confirmation, and compounds with low volatility, high molecular weight, or thermal instability may be poorly detected without derivatisation.

### 2.2 Selection of protein targets as receptors

Phytoconstituents showing favourable ADME characteristics were further evaluated by molecular docking using AutoDock Vina (PyRx 0.8) to assess their interactions with the proteins. Human pancreatic α-Amylase (PDB ID: 3OLD) and human dipeptidyl peptidase IV (DPP4) (PDB ID: 5Y7H) were obtained from the Protein Data Bank (rcsb.com/pdb database). These proteins have been selected due to their significance in postprandial glucose regulation and incretin metabolism, inflammation, and metabolic control, among other aspects of the pathophysiology of diabetes. Polar hydrogens are added, and water molecules are eliminated from the protein to enhance its refinement. The co-crystallised ligand was isolated using Discovery Studio Visualizer 2021 and saved in PDB format. The auto dock tool on the PyRx virtual screening platform was used to convert the protein PDB file to PDBQT format with the macromolecule option. 3D structure of target proteins without ligands (a) Human pancreatic α-amylase (3OLD) and (b) Human DPP IV (5Y7H) is now shown in **Figure 1**.

**Figure 1:**
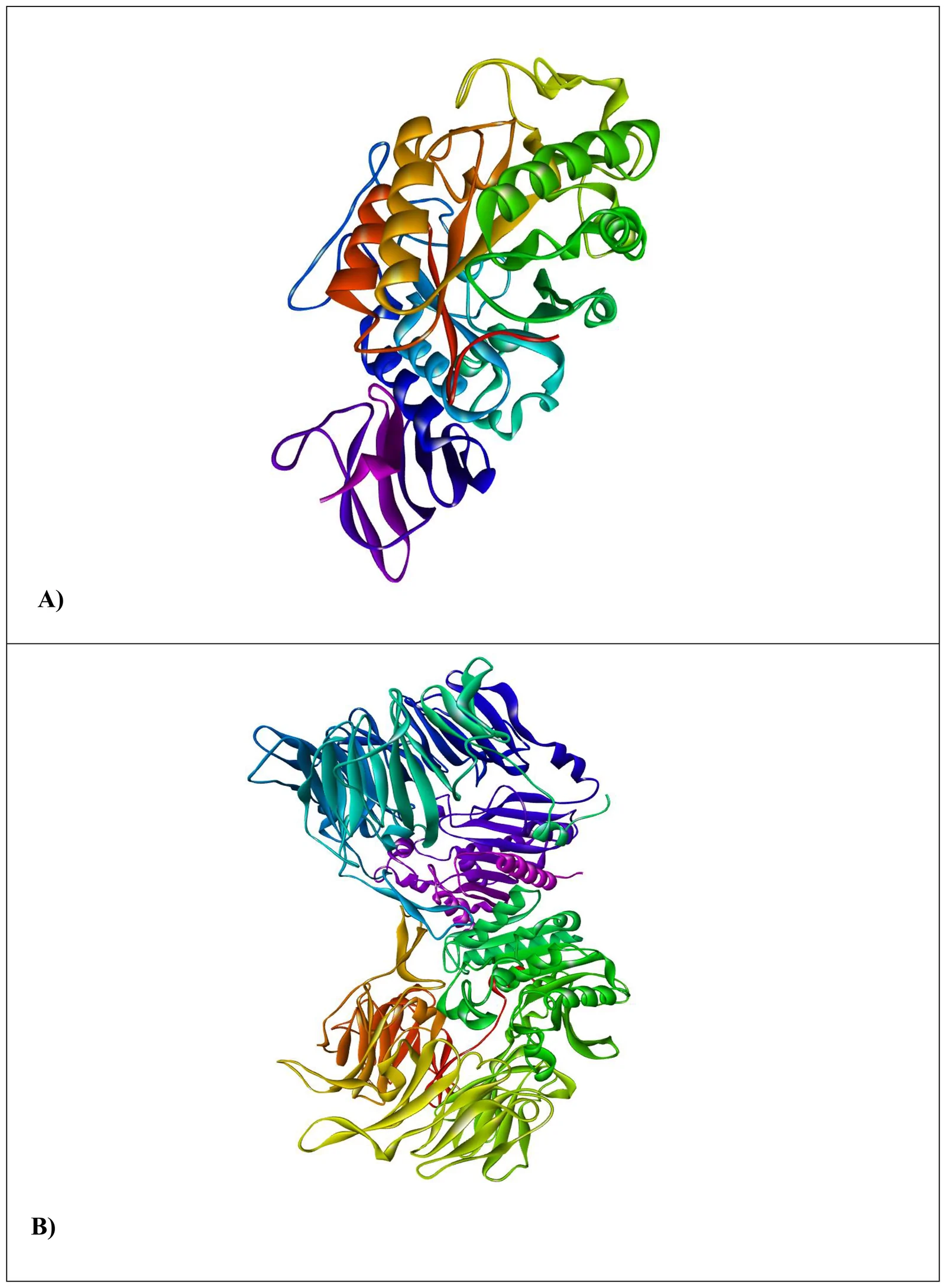
3D structure of target proteins without ligands **(a)** Human pancreatic α-amylase (3OLD) and **(b)** Human DPP IV (5Y7H).

### 2.3 Pharmacokinetic *in silico* ADME profile

Steviol (hydroxydehydrostevic acid), identified by GC–MS analysis, together with stevioside, rebaudioside A, and rebaudioside D, which are the principal steviol glycosides of *Stevia rebaudiana* reported in previous phytochemical studies (Woelwer-Rieck et al., 2010; Bergs et al., 2012; Aranda-González et al., 2014, 2015), were selected for further computational analysis because of their well-documented antidiabetic potential. The pharmacokinetic properties of these compounds were evaluated using the SwissADME web server to assess their drug-likeness and pharmacokinetic characteristics prior to molecular docking. The evaluated parameters included molecular weight, lipophilicity (LogP), hydrogen bond donors (HBD), hydrogen bond acceptors (HBA), topological polar surface area (TPSA), rotatable bonds, Lipinski’s rule of five compliance, gastrointestinal absorption, blood–brain barrier (BBB) permeability, P-glycoprotein substrate prediction, and bioavailability score (Daina et al., 2017; El Sayed et al., 2020; Al-Karmalawy et al., 2021). In this study, the *in silico* ADME analysis revealed marked differences in drug-likeness among the selected *Stevia rebaudiana* phytochemicals. Rebaudioside A, rebaudioside D, and stevioside exhibited high molecular flexibility, a greater number of hydrogen bond donors and acceptors, elevated TPSA values, and multiple Lipinski’s rule violations, suggesting comparatively lower oral bioavailability. In contrast, steviol demonstrated favourable drug-likeness, with lower TPSA, fewer hydrogen bond interactions, compliance with Lipinski’s rule of five, and a higher predicted bioavailability score, indicating more favourable pharmacokinetic properties. Overall, the ADME analysis provided comparative pharmacokinetic information for the selected compounds prior to molecular docking. Although steviol exhibited the most favourable predicted drug-likeness, the steviol glycosides were retained for molecular docking because of their established antidiabetic relevance and reported biological significance. The *in silico* ADME profile of selected phytochemicals is tabulated in **Table 2**.

**Table 2:** *In silico* ADME profile of selected phytochemicals.

S.NO	Pub ID	Ligand	RB	H-A	H-D	TPSA	LV	BBB	P-gb Sub	BAS
1	6918840	Rebaudioside A	18	23	13	374.00 A	NO - 3	NO	YES	0.11
2	71773169	Rebaudioside D	16	28	17	463.63 A	NO - 3	NO	NO	0.11
3	452967	Steviol (Hydroxydehydrostevic acid)	1	3	2	57.53 A	YES -0	YES	YES	0.85
4	442089	Stevioside	10	18	11	294.98 A	NO - 3	NO	YES	0.17

### 2.4 Selection of a suitable ligand for docking

The 3D structures of selected phytochemicals including Stevioside, Rebaudioside A, and Rebaudioside D, along with the non-volatile phytochemical, Steviol (Hydroxydehydrostevic acid), were retrieved from the PubChem database in PDB format. The ligand structures were imported into the PyRx virtual screening platform and converted to AutoDock-compatible PDBQT format using the Open Babel module prior to molecular docking. (**Figure 2**).

**Figure 2:**
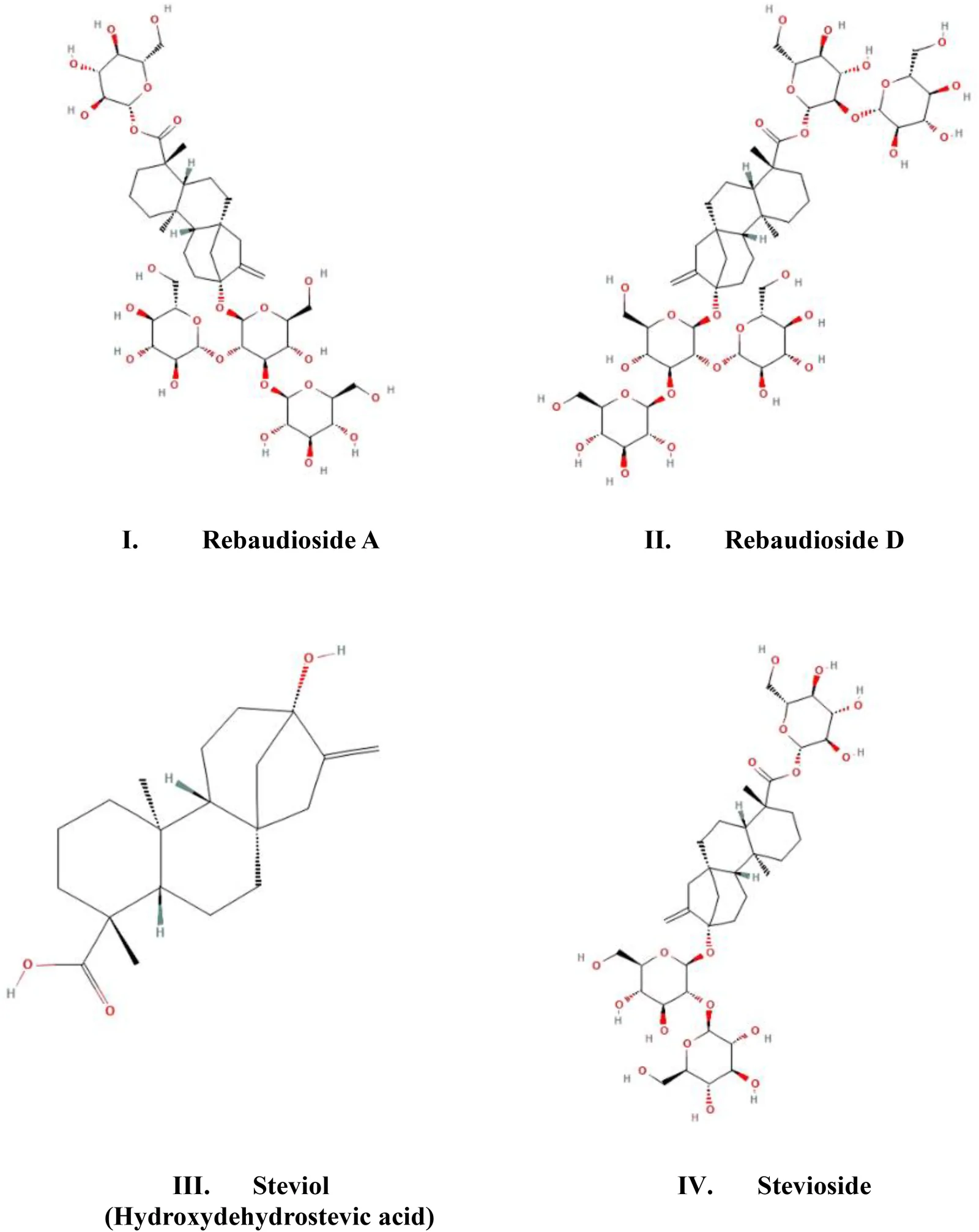
2D structures of phytochemicals used in this study (I. rebaudioside A, II. rebaudioside D, III. Steviol, IV. Stevioside).

### 2.5 Protein ligand docking

Molecular docking was performed using the Vina Wizard implemented in the PyRx virtual screening platform. The prepared macromolecule and ligand structures were converted to PDBQT format using AutoDock Tools. The binding site was defined based on the region occupied by the co-crystallized ligand and the surrounding active-site residues. For the 3OLD receptor, a grid box of 70 × 70 × 60 was used with grid-centre coordinates of x = 8.485, y = −10.645, and z = 25.372. For the 5Y7H receptor, a grid box of 80 × 80 × 80 was used with grid-center coordinates of x = 62.271, y = 88.238, and z = 35.672. The remaining docking parameters were maintained at the default Vina settings. The receptor was treated as rigid during docking, whereas ligand conformational flexibility was retained through rotation around the available rotatable bonds. Thus, the docking protocol allowed ligand flexibility while maintaining a fixed receptor conformation.

AutoDock 4.2 decomposes the predicted free energy of binding (ΔG) into several additive energy components, which are reported in **Tables 3**, **4**. The intermolecular energy represents the total non-covalent interaction energy between the ligand and receptor and comprises van der Waals, hydrogen-bonding, desolvation, and electrostatic interaction terms. The total internal energy represents the change in the ligand’s intramolecular energy between its unbound and docked conformations, reflecting the conformational strain incurred upon binding. The torsional free energy represents an entropic penalty proportional to the number of active rotatable bonds in the ligand, accounting for the loss of conformational freedom upon binding. The estimated free energy of binding (ΔG) is calculated as the sum of the intermolecular energy and the torsional free energy, using the empirical scoring function implemented in AutoDock 4.2. The estimated inhibition constant (Ki) is subsequently calculated from the predicted binding free energy using the standard thermodynamic relationship implemented in AutoDock 4.2. The docking outcomes were visualised using Discovery Studio 2021 Client with the presentation of the interaction patterns and the binding affinities, such as the amino acid interaction, hydrogen and hydrophobic bonds. All docking positions had been depicted both in 2D and 3D format and the amino acid residues that promote the docking of particular plant compounds to target proteins were shown. Compounds exhibiting more favourable predicted binding energies were considered for subsequent comparative analysis. The predicted binding energies were interpreted as relative estimates of protein–ligand interaction and not as experimentally validated measures of inhibitory activity (Ahmad et al., 2017). The binding poses and interactions were visualised using Discovery Studio Visualizer 2021.

**Table 3:** Statistical data of molecular docking of the human pancreatic α-amylase and the identified phytocompounds complex.

Parameter	Rebaudioside A	Rebaudioside D	Steviol	Stevioside
Binding energy	-4.28	-8.24	-8.46	-6.36
Ligand efficiency	-0.06	0.15	-0.37	-0.11
Inhib constant	729.57	905.44	629.81	21.87
Inhib constant units	µM	nM	nM	µM
Intermolecular energy	-12.33	-14.51	-9.35	-12.62
Vdw hb desolv energy	-12.26	-13.76	-8.77	-12.42
Electrostatic energy	-0.07	0.75	-0.59	-0.2
Total internal energy	-12.4	-9.07	0	-9.51
Torsional energy	8.05	6.26	0.89	-6.26
Unbound energy	-12.4	-9.07	0	-9.51

All energy values are expressed in kcal/mol and were generated using AutoDock 4.2. The estimated inhibition constant (Ki) was calculated from the predicted binding free energy (ΔG) and represents a computational estimate rather than an experimentally determined value. Ligand efficiency, binding energy normalized to heavy-atom count (Hopkins et al., 2004), was therefore included to facilitate comparison among ligands differing in molecular size.

**Table 4:** Statistical data of molecular docking of the DPP IV and the identified phytocompounds complex.

Parameter	Rebaudioside A	Rebaudioside D	Steviol	Stevioside
Binding energy	-8.34	-5.95	-4.42	-6.33
Ligand efficiency	-0.36	-0.11	-0.07	-0.11
Inhib constant	768.72	43.37	579.87	22.92
Inhib constant units	nM	uM	µM	µM
Intermolecular energy	-9.24	-12.22	-12.47	-12.59
Vdw hb desolv energy	-8.84	-11.9	-12.37	-12.42
Electrostatic energy	-0.4	-0.31	-0.1	-0.17
Total internal energy	0.02	-9.69	-15.6	-8.41
Torsional energy	0.89	-6.26	-6.05	-6.26
Unbound energy	0.02	-9.69	-15.6	-8.41

All energy values are expressed in kcal/mol and were generated using AutoDock 4.2. The estimated inhibition constant (Ki) was calculated from the predicted binding free energy (ΔG) and represents a computational estimate rather than an experimentally determined value. Ligand efficiency, binding energy normalized to heavy-atom count (Hopkins et al., 2004), was therefore included to facilitate comparison among ligands differing in molecular size.

### 2.6 Molecular dynamics simulation

The interactive behaviour and dynamic stability of the protein-ligand complexes were studied through the GROMACS package, employed in molecular dynamics (MD) simulations to evaluate the dynamic behaviour of the protein–ligand complexes beyond static docking predictions. RMSD was employed to compare the stability of backbones on a global scale, RMSF was utilised to determine the residue-level bending, radius of gyration (Rg) was employed to examine the compact nature of the protein, and solvent-accessible surface area (SASA) was used to determine alterations in solvent exposure throughout the simulation. MM-PBSA analysis was subsequently performed to estimate the binding free energy of the protein–ligand complexes and evaluate the energetic contributions governing ligand binding. These analyses were used to characterise the structural stability, conformational flexibility, compactness, solvent accessibility, and predicted binding energetics of the protein–ligand complexes throughout the simulation.

Molecular dynamics simulations were performed for the steviol–α-amylase and Rebaudioside A–DPP-IV complexes using GROMACS 2023.1. The systems were prepared using CHARMM-GUI v3.7/CHARMM force-field conversion, solvated with TIP3P water, and neutralized with Na^+^/Cl^-^ ions. Energy minimization, equilibration, and NPT equilibration were performed prior to production MD. A 2 fs timestep was used, with PME for long-range electrostatic interactions and a Verlet cutoff scheme with 1.2 nm Coulombic and van der Waals cutoffs. Temperature was maintained at 310.15 K using the V-rescale thermostat (τT = 1.0 ps), while pressure was maintained at 1 bar using isotropic C-rescale pressure coupling (τP = 5.0 ps). LINCS was used to constrain hydrogen-containing bonds, with periodic boundary conditions in all three directions (xyz).

MD simulations were performed as single 100 ns production runs per complex, using default GROMACS simulation parameters, without replicate trajectories or a ligand-free (apo) protein control. Principal component analysis (PCA) and free-energy landscape (FEL) analyses were performed to assess the major collective motions and conformational states of the protein–ligand complexes. In addition to RMSD, RMSF, radius of gyration and SASA analyses, principal component analysis (PCA), free-energy landscape (FEL) analysis, hydrogen-bond analysis, dynamic cross-correlation analysis (DCCM), and per-residue MM-PBSA energy decomposition were performed to further characterise the conformational dynamics, intermolecular interactions, correlated motions, and residue-level energetic contributions of the selected protein–ligand complexes.

## 3 Results and discussion

### 3.1 GC–MS metabolite profiling of *Stevia rebaudiana*

The GC-MS analysis identified 24 compounds with distinct molecular masses ranging from 102.13 to 468.8 g/mol, as detailed in **Table 5**. The GC-MS analysis of *Stevia rebaudiana* identified a wide variety of compounds, such as phenolics, glycosides, fatty acids, carbohydrates, and more. The identified significant compounds include phenolics like methyl salicylate, 3-allyl-6-methoxyphenol, orcinol, β-ionone, and alloaromadendrene oxide; glycosides such as ethyl α-d-glucopyranoside, d-gluconic acid, and γ lactone; fatty acids including e-15-heptadecenal, 9,12,15-octadecatrienoic acid and n-hexadecanoic acid; carbohydrates like ribitol and d-allose; and other phytochemicals including ethane, olean-12-en-3-ol, 1,1-diethoxy, butanoic acid, 3-methyl, acetate, neophytadiene, sclareol, 1,7-dimethylxanthine, hydroxydehydrostevic acid (steviol), stigmasterol, γ-sitosterol, lup-20(29)-en-3-ol, β-amyrin and β-amyrone. The diverse metabolite profile identified in the present study is generally consistent with previous GC–MS investigations of *Stevia rebaudiana*, which have reported the presence of phenolics, terpenoids, sterols, fatty acids, and steviol-related metabolites, highlighting the rich phytochemical composition of the species. Although GC–MS provides valuable metabolite profiling, it primarily detects volatile and semi-volatile compounds, and compound identification is based on spectral library matching; therefore, complementary analytical techniques such as HPLC or LC–MS may be required for comprehensive characterization of specific phytochemicals, particularly steviol glycosides.

**Table 5:** List of phytochemicals present in GC-MS analysis of stevia leaves.

S.No	Peak	Area %	Compound name	Formula	Mass
1	2.747	1.06	Ethane, 1,1-diethoxy-	C_6_H_14_O_2_	118.17
2	4.051	0.41	Butanoic acid, 3-methyl-	C_5_H_10_O_2_	102.13
3	7.735	0.39	Methyl salicylate	C_6_H_8_O_3_	152.15
4	8.117	0.69	Ribitol	C_5_H_12_O_5_	152.15
5	9.243	0.2	3-Allyl-6-methoxyphenol	C_10_H_12_O_2_	164.2
6	10.712	8.11	D-Allose	C_6_H_12_O_6_	180.16
7	11.425	0.39	D-Gluconic acid, γ lactone	C_6_H_10_O_6_	178.14
8	11.743	0.57	Ethyl α-d-glucopyranoside	C_8_H_16_O_6_	208.21
9	12.736	0.52	Orcinol	C_7_H_8_O_2_	124.14
10	12.952	0.95	Neophytadiene	C_20_H_38_	278.5
11	12.766	3.42	n-Hexadecanoic acid	C_16_H_32_O_2_	256.42
12	14.555	0.55	E-15-Heptadecenal	C_17_H_32_O	252.4
13	14.988	7.25	9,12,15-Octadecatrienoic acid	C_18_H_30_O_2_	278.4
14	15.872	3.71	Sclareol	C_20_H_36_O_2_	308.5
15	16.298	0.52	1,7-Dimethylxanthine	C_7_H_8_N_4_O_2_	180.16
16	16.61	1.29	β-Ionone	C_13_H_20_O	192.3
17	19.282	2.68	Alloaromadendrene oxide	C_15_H_24_O	220.35
18	20.434	12.23	Hydroxydehydrostevic acid (Steviol)	C_20_H_30_O_3_	318.4
19	25.377	1	Stigmasterol	C_29_H_48_O	412.7
20	25.937	1.09	γ - Sitosterol	C_29_H_50_O	414.7
21	27.514	0.51	Olean-12-en-3-ol, acetate	C_32_H_52_O_2_	468.8
22	27.514	0.51	β - Amyrin	C_30_H_50_O	426.7
23	27.514	0.51	β-Amyrone	C_30_H_48_O	424.7
24	28.055	1.41	Lup-20(29)-en-3-ol, acetate	C_32_H_52_O_2_	468.8

### 3.2 *In silico* ADME analysis of selected phytochemicals

The *in silico* ADME assessment revealed pronounced differences in physicochemical and pharmacokinetic properties among the selected phytochemicals. Rebaudioside D exhibited the highest topological polar surface area (TPSA) (463.63 Å²), followed by rebaudioside A (374.00 Å²) and stevioside (294.98 Å²), whereas steviol showed the lowest TPSA (57.53 Å²) and the fewest hydrogen bond donors and acceptors (HBD = 2; HBA = 3), consistent with its comparatively lower molecular flexibility (RB = 1) than rebaudioside A (RB = 18) and rebaudioside D (RB = 16). Steviol satisfied all five of Lipinski’s rule of five criteria, whereas rebaudioside A, rebaudioside D, and stevioside each violated three criteria, suggesting comparatively less favourable predicted oral pharmacokinetic properties associated with their larger molecular size and higher polarity. These predicted pharmacokinetic characteristics should be interpreted together with the molecular docking results and the biological roles of the target proteins rather than as direct measures of antidiabetic activity.

Predictions of blood–brain barrier (BBB) permeability showed that steviol alone was BBB-permeable, while all three glycosylated compounds were predicted to be BBB-impermeable. This distinction, however, has limited physiological relevance to the present study, since neither α-amylase nor DPP-IV is a central nervous system target. The comparatively high TPSA and lower predicted systemic absorption of the steviol glycosides may favour their retention within the gastrointestinal lumen, where α-amylase-mediated carbohydrate digestion occurs, although this hypothesis requires experimental pharmacokinetic validation. Such a pharmacokinetic profile is broadly consistent with the localized mode of action reported for clinically used α-amylase inhibitors such as acarbose (Xu et al., 2020). In contrast, the comparatively favourable predicted absorption characteristics and higher bioavailability score of steviol may be advantageous for interactions with DPP-IV, which exists as both membrane-bound and soluble forms in peripheral tissues and systemic circulation. Overall, the observed ADME differences between steviol and the steviol glycosides should be interpreted as predicted pharmacokinetic characteristics that may influence target accessibility rather than as direct indicators of antidiabetic efficacy.

### 3.3 Protein ligand docking

All the selected compounds were docked against the active pocket sites of human pancreatic α-amylase and DPP IV using AutoDock 4.2. These binding strengths of the compounds to the target proteins are shown, and the 2D interaction of phytochemicals and standard drugs against α-amylase and DPP IV were presented in **Figures 3A, B**, respectively. The identified compounds exhibited stronger binding interactions, with values ranging from -4.28 to -8.46 kcal/mol for 3OLD and -4.42 to -8.34 kcal/mol for 5Y7H.

**Figure 3:**
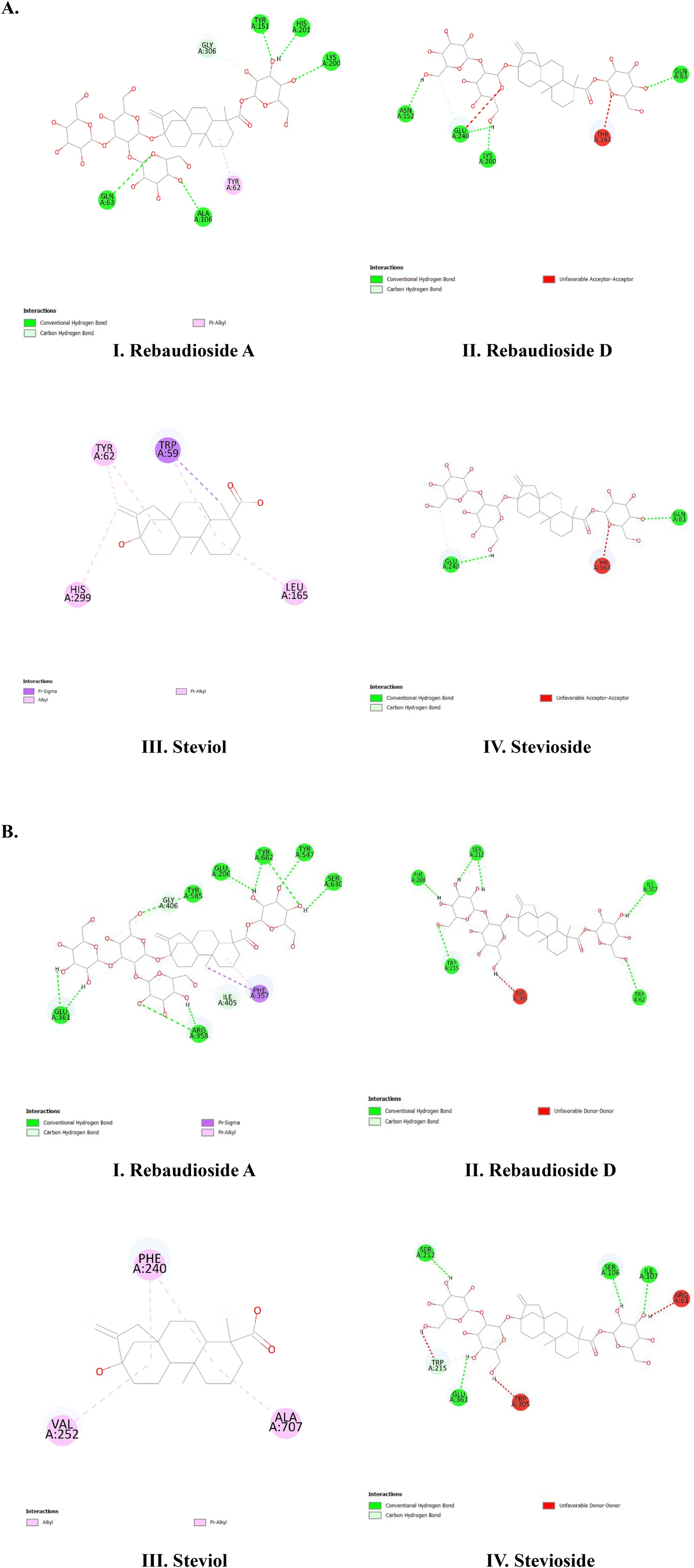
**(A)** 2D interaction of phytochemicals against human pancreatic α-amylase (I. rebaudioside A, II. rebaudioside D, III. steviol, IV. stevioside). **(B)** 2D interaction of phytochemicals against human DPP IV (I. rebaudioside A, II. rebaudioside D, III. steviol, IV. stevioside).

#### 3.3.1 Molecular docking analysis with human pancreatic α-amylase

The molecular docking study against human pancreatic α-amylase revealed clear differences in predicted binding energy among the four phytocompounds evaluated (**Figure 3A**). Steviol showed the most favourable predicted binding energy (−8.46 kcal/mol), closely followed by rebaudioside D (−8.24 kcal/mol), while stevioside (−6.36 kcal/mol) and rebaudioside A (−4.28 kcal/mol) showed comparatively weaker predicted binding. Because steviol and the steviol glycosides differ substantially in molecular size and flexibility, ligand efficiency (binding energy normalized to heavy-atom count) was also considered to provide a size-corrected comparison. By this measure, steviol showed the most favourable ligand efficiency (−0.37), consistent with efficient use of its compact scaffold, whereas rebaudioside A showed the lowest ligand efficiency (−0.06), suggesting that its large glycosylated structure is not proportionately well accommodated within the α-amylase active site. The comparatively lower ligand efficiency observed for the glycosylated steviol derivatives is likely influenced by their larger molecular size, higher flexibility, and increased polarity, which may reduce the efficiency with which individual heavy atoms contribute to the overall predicted binding affinity. The predicted inhibition constants (Ki) followed a similar pattern: steviol showed the lowest predicted Ki (629.81 nM), suggesting comparatively stronger predicted binding affinity toward human pancreatic α-amylase, and rebaudioside D also fell within the nanomolar range (905.44 nM), whereas rebaudioside A (729.57 µM) and stevioside (21.87 µM) showed substantially weaker predicted inhibition, in line with their less favourable binding energies. As these Ki values are docking-derived estimates rather than experimentally measured constants, they should be interpreted as a relative ranking of predicted potency rather than as quantitative measures of actual enzyme inhibition.

Energy component analysis indicated that van der Waals, hydrogen-bonding, and desolvation terms were the dominant contributors to predicted binding across all four compounds. Rebaudioside D showed the most negative intermolecular energy (−14.51 kcal/mol) and vdW + H-bond + desolvation energy (−13.76 kcal/mol), indicating more favourable predicted intermolecular interactions within the docking pose; however, this was offset by a comparatively high torsional energy (6.26 kcal/mol), which may be associated with the conformational flexibility of the glycosylated structure. Steviol, by contrast, showed a much lower torsional energy (0.89 kcal/mol), consistent with a more energetically stable binding pose. The human pancreatic α-amylase active site is a relatively compact binding pocket lined by residues such as Trp58, Trp59, Tyr62, and His299. Overall, steviol exhibited the most favourable predicted docking profile among the evaluated phytocompounds based on the combined interpretation of predicted binding energy, estimated Ki, ligand efficiency, and docking energy components. Consequently, steviol was selected for molecular dynamics simulations to further evaluate the dynamic behaviour of the protein–ligand complex under simulated conditions. It should therefore be noted that direct comparison of docking scores among structurally diverse phytochemicals should be interpreted with caution, as molecular size, conformational flexibility, and polarity can influence docking-derived binding energies independently of true biological affinity. Statistical data from the molecular docking of human pancreatic α-amylase with the selected phytocompounds are presented in **Table 3**.

#### 3.3.2 Molecular docking analysis with DPP-IV

Docking analysis against DPP-IV revealed a different binding preference pattern compared to α-amylase (**Figure 3B**), consistent with the more hydrogen-bond-rich, polar character of the DPP-IV active site. Among the four compounds, rebaudioside A showed the most favourable predicted binding energy (−8.34 kcal/mol), followed by stevioside (−6.33 kcal/mol) and rebaudioside D (−5.95 kcal/mol), while steviol showed the least favourable binding energy against DPP-IV (−4.42 kcal/mol). Ligand efficiency followed a comparable trend, with rebaudioside A showing the most favourable value (−0.36) and steviol the least favourable (−0.07). This lower ligand efficiency suggests comparatively less efficient utilization of the binding interactions by steviol within the DPP-IV binding pocket, rather than a complete absence of interaction, since steviol still showed a substantial negative intermolecular energy term (−12.47 kcal/mol).

The predicted inhibition constants (Ki) of rebaudioside A showed the lowest predicted Ki (768.72 nM), within the nanomolar range, while rebaudioside D (43.37 µM), steviol (579.87 µM), and stevioside (22.92 µM) showed higher predicted Ki values, suggesting comparatively lower predicted binding affinity toward DPP-IV. As with the α-amylase analysis, these Ki values are docking-derived and should be interpreted as a relative ranking rather than as measured inhibition constants. Overall, rebaudioside A exhibited the most favourable docking profile among the evaluated phytocompounds, as indicated by its predicted binding affinity, estimated inhibition constant, and ligand efficiency. These findings suggest that rebaudioside A may represent a promising candidate for DPP-IV inhibition. Consequently, rebaudioside A was selected for subsequent molecular dynamics simulations to further evaluate the stability of the protein–ligand complex under dynamic conditions. Statistical data from the molecular docking of DPP-IV with the selected phytocompounds are presented in **Table 4**.

#### 3.3.3 Comparative interpretation of α-amylase and DPP-IV docking results

A comparative evaluation of both docking studies highlights a target-specific binding preference among *Stevia rebaudiana* phytochemicals. Steviol showed a more favourable predicted docking profile toward α-amylase, whereas rebaudioside A exhibited more favourable predicted interaction with DPP-IV. This discrimination is explained by the differences in active site structure and active ligand physicochemical characteristics. Interaction of Steviol against human pancreatic α-amylase and Rebaudioside A against DPPIV and their Ligplot+ interaction are shown in **Figures 4A, B**. The docking results suggest that the α-amylase binding pocket may favour smaller, less flexible structures like steviol that experienced fewer torsional penalties and made stable complexes with hydrophobic and van der Waals forces. Conversely, the DPP-IV binding pocket appeared more accommodating toward a larger-sized glycosylated structure, which enabled rebaudioside A to establish extensive hydrogen bonding and stabilise in the binding pocket of the enzyme. Based on the combined interpretation of predicted binding energy, estimated inhibition constant (Ki), ligand efficiency, and docking energy parameters, the steviol–α-amylase and rebaudioside A–DPP-IV complexes were selected for subsequent molecular dynamics simulations to evaluate their dynamic behaviour under simulated conditions. The MD simulations (RMSD, RMSF, radius of gyration, and SASA) were performed to further evaluate the dynamic behaviour of the selected complexes under simulated conditions.

**Figure 4:**
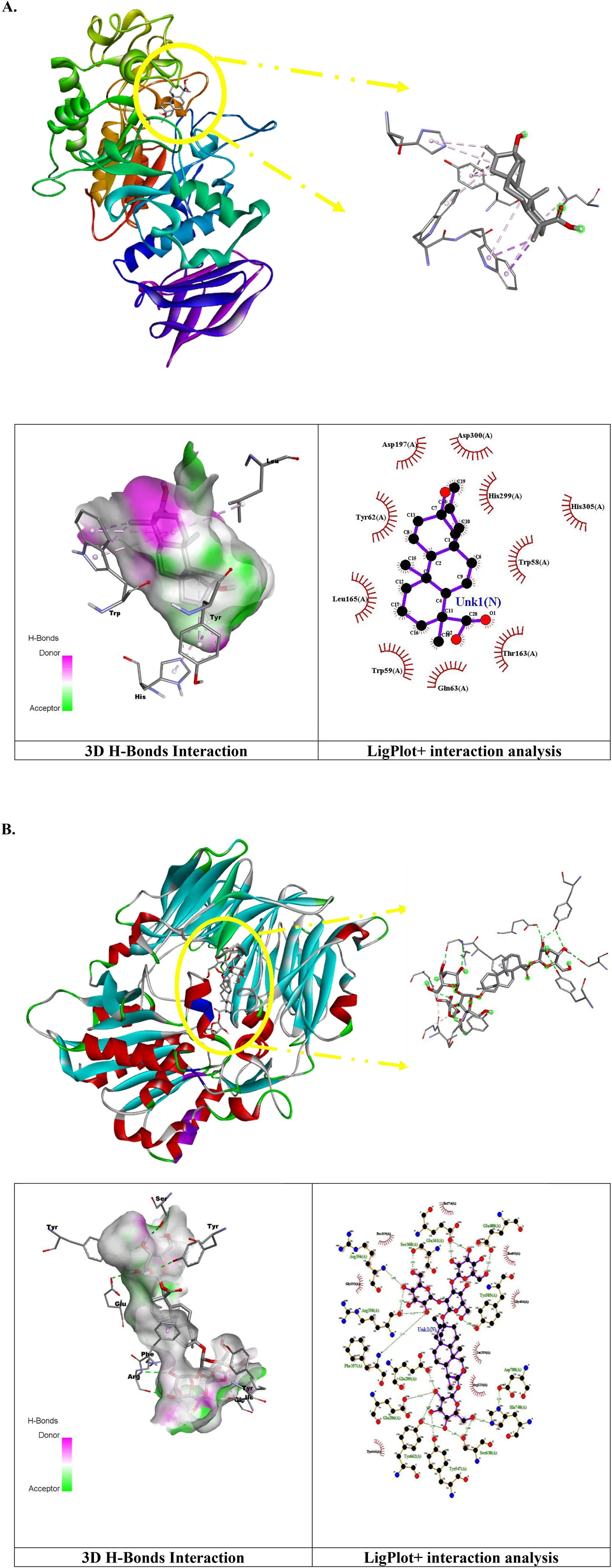
**(A)** Interaction of steviol against human pancreatic α-amylase. **(B)** Interaction of rebaudioside A against human DPP IV.

### 3.4 Hydrogen bonds interaction

#### 3.4.1 Hydrogen bond interaction analysis with human pancreatic α-amylase

The analysis of the Hydrogen bond interaction of the α-amylase-ligand complexes (**Table 6**) showed that there were different binding patterns among the various phytocompounds that were investigated. Rebaudioside A had the maximum number of hydrogen bonds (5) with key residues of GLN63, ALA106, TYR151, HIS201, and LYS200, although the binding affinity (-4.28 kcal/mol) was lowest of the rest compounds tested. This implies that increased hydrogen bonds may not always be related to increased binding strength, especially in cases of too much molecular flexibility and steric constraints. Rebaudioside D (−8.24 kcal/mol) formed four hydrogen bonds with ASN152, GLU240, LYS200, and GLN63, indicating that both hydrogen bonding and other non-covalent interactions contributed to the predicted docking pose. Stevioside, with a medium binding affinity of (−6.36 kcal/mol), was able to make two hydrogen bonds with GLU240 and GLN63, suggesting a contribution of polar interactions to the predicted docking pose. However, steviol had the highest binding affinity to α-amylase (-8.46 kcal/mol) even though it did not form any conventional hydrogen bonds. These observations suggest that favourable docking of steviol may be influenced predominantly by non-polar interactions rather than conventional hydrogen bonding.

**Table 6:** Hydrogen bond interactions of the human pancreatic α-amylase and identified phytocompounds complex.

S. No	Pub ID	Ligand x 3OLD	Binding affinity (kcal/mol)	No. Of H bonds	Amino acids involved in H bonding
1	6918840	Rebaudioside A	-4.28	5	GLN 63, ALA 106, TYR 151, HIS 201, LYS 200
2	71773169	Rebaudioside D	-8.24	4	ASN 152, GLU 240, LYS 200, GLN 63
3	452967	Steviol (Hydroxydehydrostevic acid)	-8.46	–	–
4	442089	Stevioside	-6.36	2	GLU 240, GLN 63

#### 3.4.2 Hydrogen bond interaction analysis with DPP-IV

As compared to α-amylase, DPP-IV demonstrated a high reliance on hydrogen bonding to stabilize ligands. Rebaudioside A had the best binding affinity (−8.34 kcal/mol) and the maximum number of hydrogen bonds (7) with essential active-site residues, such as GLU361, ARG358, TYR585, GLU206, TYR662, TYR547, and SER630. These residues are located within the predicted binding pocket and may contribute to the observed docking pose of rebaudioside A. Rebaudioside D, which had a binding energy of -5.95 kcal/mol, formed five hydrogen bonds with PHE208, TRP215, SER212, ILE107, and TRP62, indicating moderate interaction stability. Stevioside, with a binding affinity of -6.33 kcal/mol, had four hydrogen bonds with SER212, GLU261, SER106, and ILE107. These residues are located within the predicted binding pocket and may contribute to the observed docking pose of rebaudioside A. As with α-amylase, steviol exhibited the weakest predicted binding affinity toward DPP-IV (−4.42 kcal/mol), suggesting comparatively less favourable interactions with the DPP-IV binding pocket.

A LigPlot + analysis of steviol in the complex with human pancreatic α-amylase has shown that the interaction is stabilised by mostly hydrophobic contacts as opposed to hydrogen bonds. Steviol made π-alkyl and π-σ interactions with active-site residues, such as Trp59, Tyr62, Leu165, His299 and Trp58. The lack of the usual hydrogen bonds indicates that the van der Waals and hydrophobic forces have a significant contribution to the stability of the steviol-α-amylase complex, which accounts for the high binding affinity as indicated in the docking experiment. LigPlot interaction analysis revealed that rebaudioside A affinity binds in the active site of DPP-IV via a large number of hydrogen bonds. It formed several hydrogen bonds with critical residues, such as Glu361, Arg358, Tyr585, Glu206, Tyr662, Tyr547, and Ser630, among other hydrophobic interactions. The high number of hydrogen bonds explains the strong binding affinity of rebaudioside A toward DPP-IV observed in the docking analysis and supports its selection for further molecular dynamics simulations.

### 3.5 MD Simulation: molecular dynamics parameters (RMSD, RMSF, Rg, and SASA) of selected complexes.

Molecular dynamics (MD) simulations were performed on the selected steviol–α-amylase and rebaudioside A–DPP-IV complexes to evaluate their conformational behaviour beyond the static molecular docking predictions. Structural behaviour during the 100 ns simulations was assessed using root mean square deviation (RMSD), root mean square fluctuation (RMSF), radius of gyration (Rg), and solvent-accessible surface area (SASA). The corresponding MD trajectories are presented in **Figure 5A, B**.

**Figure 5:**
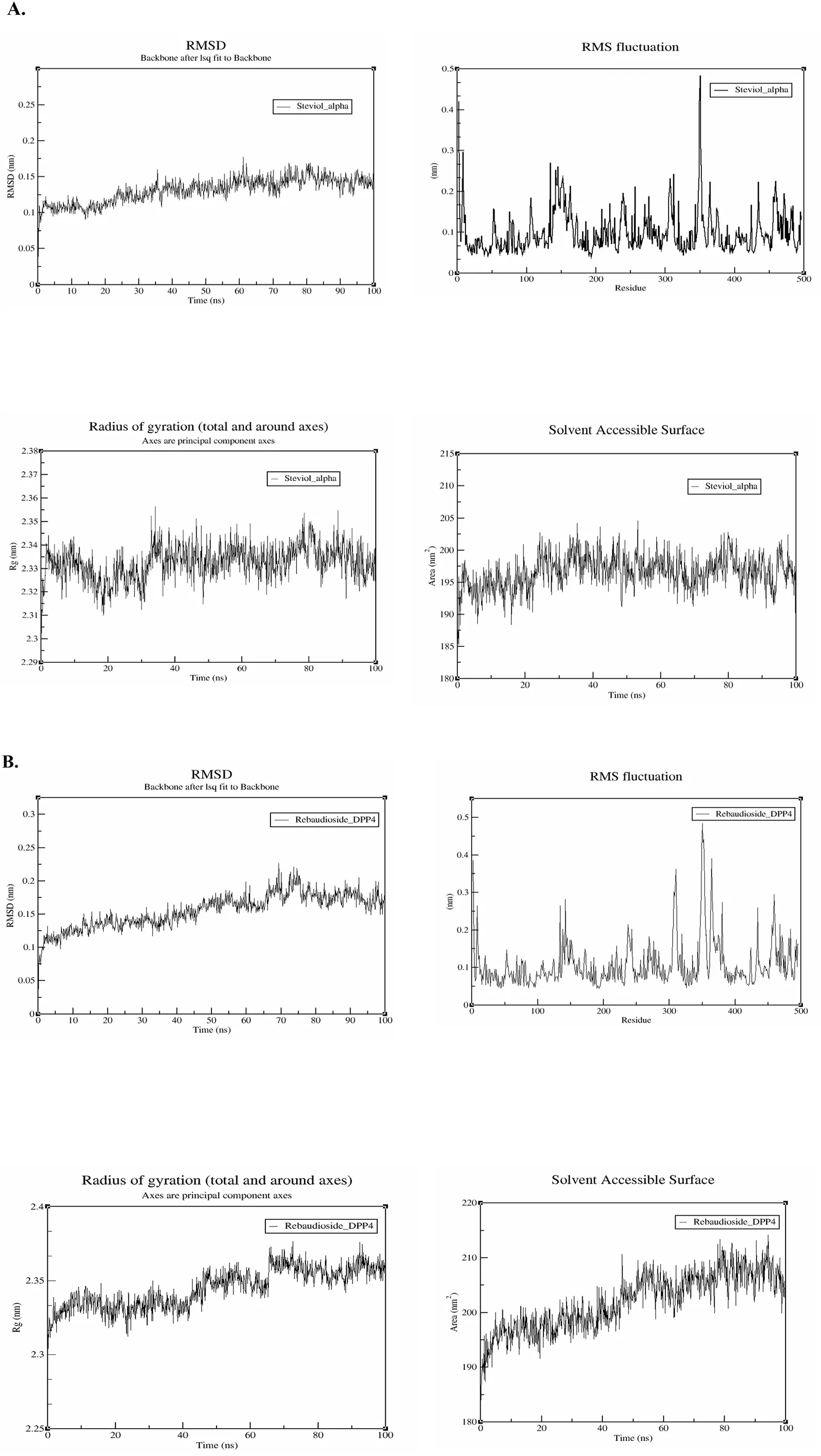
**(A)** RMSD, RMS fluctuation, radius of gyration and solvent accessible surface of steviol against human pancreatic α-amylase. **(B)** RMSD, RMS fluctuation, radius of gyration and solvent accessible surface of rebaudioside A against human DPP IV .

#### 3.5.1 Root mean square deviation (RMSD)

The backbone RMSD of the α-amylase–steviol complex showed a gradual, sustained upward trend over the course of the 100 ns trajectory, rising from an average of approximately 0.10–0.11 nm in the first 20 ns to approximately 0.14–0.15 nm by 80–90 ns, with only modest signs of levelling off in the final 10 ns. The RMSD values ranged from approximately 0.09 to 0.18 nm over the 100 ns simulation, with an overall average of 0.13 nm, and a transient maximum of approximately 0.18 nm observed near 61 ns. While RMSD values remained below 0.2 nm throughout, the sustained upward drift suggests some degree of conformational adjustment of the backbone over the course of the simulation, despite the absence of conventional hydrogen bonding between steviol and α-amylase. In the DPP-IV–rebaudioside A complex, the backbone RMSD showed a gradual upward trend through the simulation, rising from an average of approximately 0.13 nm in the first 20 ns to approximately 0.15–0.17 nm by 40–60 ns, followed by a more pronounced increase to a transient peak of approximately 0.22 nm around 65–75 ns. RMSD values partially declined after this peak but stabilised at a new plateau of approximately 0.17–0.18 nm for the remainder of the simulation, rather than returning to earlier baseline levels. The overall average RMSD (excluding the initial equilibration period) was approximately 0.16 nm, modestly higher than that observed for the steviol–α-amylase system.

The RMSD values for both protein–ligand complexes remained below 0.25 nm throughout the 100 ns simulation, suggesting overall structural stability during the production run. The steviol–α-amylase complex exhibited comparatively lower RMSD values than the rebaudioside A–DPP-IV complex, suggesting smaller deviations from the initial structure, whereas the rebaudioside A–DPP-IV complex displayed moderately greater structural fluctuations. These observations are broadly consistent with the molecular docking results and indicate that both complexes remained structurally stable under the simulated conditions. However, these findings are based on a single 100 ns production simulation for each complex and should be interpreted with appropriate caution, as replicate trajectories and ligand-free (apo) protein simulations were not performed.

### 3.5.2 Root mean square fluctuation

Root Mean Square Fluctuation (RMSF) is employed to measure local conformational changes along the protein chain and also to investigate the changes in the position of ligand atoms at a particular temperature and pressure. With increased RMSF values, there is increased flexibility in the structure, especially in areas like loops and turns. The RMSF profile of the steviol complexed with the α-amylase backbone reveals that the majority of the residues have small fluctuations by an average of between 0.05 and 0.12 nm, which indicates limited backbone flexibility across most residues during simulation. The increased variations are restricted principally to certain regions, where moderate peaks to near 0.20 -0.27 nm occur around 120–160 and 280–320 residues that probably represent flexible loop regions or surface-exposed segments. There is a sharp peak near 0.45–0.48 nm around residue 350, indicating a tendency towards local flexibility in a terminal or loop structure unrelated to binding the ligand. A comparably large fluctuation was also observed at the N-terminal residues (residues 2–9, peaking at 0.42 nm at residue 2), consistent with the inherently high flexibility typically observed at unrestrained protein termini rather than indicating any ligand-related effect. The residues around the binding location are not very dynamic, which is why the interaction between steviol and α-amylase is maintained during the simulation.

In the case of the DPP-IV rebaudioside A complex, RMSF values of most of the residues are in the 0.06-0.15nm range, which denotes overall structural stability. Different extents of fluctuation are identified between residues 300 and 360, where the maximum values are observed to be about 0.45nm, which indicates that the loop or hinge regions of the DPP-IV are more flexible. As with the α-amylase complex, comparably high fluctuation was also observed at the N-terminal residues (residue 2, RMSF 0.39 nm), consistent with the expected flexibility of unrestrained protein termini rather than a ligand-related effect. There are also small variations (approximately 0.20-0.30 nm) in the range of residues 140–170 and 420- 460. Despite these localised motions, residues associated with the ligand-binding region show limited fluctuations, suggesting comparatively limited fluctuations around the predicted binding region. Both complexes display low RMSF values across most residues, indicating structural stability of α-amylase and DPP-IV during the molecular dynamics simulations. The observed high-fluctuation regions are limited to flexible loops and terminal segments, which is typical for large enzymes. The comparatively small oscillations around the active sites are consistent with the docking results and are consistent with limited fluctuations around the predicted binding regions under the simulated conditions.

### 3.5.3 Radius of gyration

The radius of gyration (Rg) indicates that both protein–ligand complexes maintained overall structural compactness throughout the 100 ns molecular dynamics simulation. The Rg values in the steviol-α-amylase complex were also quite constant, with a range of values between 2.31 and 2.35 nm with an average of about 2.33 nm, indicating that the binding of the ligand did not cause much unfolding or unfolding of the structure of the protein. A few deviations that occur at the beginning of the simulation are indicative of normal backbone relaxation. On the other hand, the rebaudioside A -DPP-IV complex exhibited slightly higher values of Rg with a gradual rise beyond some 40 nse followed by a decelerating rise that approached, but had not fully reached, a stable plateau by the end of the simulation. The limited Rg variation observed in both systems suggests that the proteins remained compact and did not undergo major structural rearrangement in the presence of their respective ligands, remaining broadly consistent with the docking poses obtained prior to simulation.

#### 3.5.4 Solvent-accessible surface area

The solvent-accessible surface area (SASA) analysis reveals that the solvent exposure of both the protein-ligand complexes remained constant throughout the 100 ns molecular dynamics simulation. The SASA values of the steviol–α–amylase ranged within a very small range of about 190–202 nm² with an average of about 196–198 nm², indicating that the surface exposure of the protein and its overall structure did not change significantly when it bound the ligand. Minor oscillations observed throughout the trajectory reflect normal backbone breathing motions rather than structural disruption. The rebaudioside A–DPP-IV complex showed a higher overall SASA than the steviol–α-amylase complex, with values ranging from approximately 182 to 213 nm² and a mean of approximately 202 nm². Unlike the steviol–α-amylase complex, SASA in this system showed a continuous, gradual increase across the entire 100 ns trajectory rather than reaching a stable plateau, rising from an average of approximately 195 nm² in the first 20 ns to approximately 207 nm² by 80–100 ns. This sustained upward trend suggests that the complex may not have reached full surface-area equilibrium within the simulated timeframe. While the steviol–α-amylase complex reached a relatively stable SASA profile, the sustained rise observed for the rebaudioside A–DPP-IV complex indicates that surface exposure had not yet stabilised by the end of the 100 ns simulation. These molecular dynamics analyses are based on a single independent 100 ns production simulation for each protein–ligand complex, without replicate simulations or a ligand-free (apo) protein control performed under identical conditions. The RMSD, RMSF, radius of gyration (Rg), and solvent-accessible surface area (SASA) profiles should be interpreted as indicative of the conformational behaviour of the complexes under the simulated conditions rather than as definitive evidence of their dynamic stability. Additional independent simulation replicates and apo-protein simulations would be required to assess the reproducibility and robustness of these observations.

#### 3.5.5 Principal component analysis and free-energy landscape analysis

Principal component analysis (PCA) was performed to characterize the major collective motions sampled by the protein–ligand complexes during the molecular dynamics simulations. The corresponding free-energy landscapes projected onto PC1 and PC2 revealed distinct low-energy basins for both the steviol–α-amylase and rebaudioside A–DPP-IV complexes (**Figure 6**). These low-energy regions represent energetically favourable conformational states sampled during the simulations, while the presence of multiple basins indicates transitions among different conformational states. Overall, the PCA-derived free-energy landscapes provide additional insight into the conformational behaviour of the complexes beyond RMSD, RMSF, radius of gyration, and SASA analyses (**Table 7**). These findings should be interpreted as computational observations under the simulated conditions and do not independently establish experimental binding stability or inhibitory activity. Residue-wise MM-PBSA energy decomposition was performed to identify the amino acid residues contributing to the predicted binding energetics of the steviol–α-amylase and rebaudioside A–DPP-IV complexes. Principal component analysis and MM-PBSA binding free energy calculations were subsequently performed to assess conformational dynamics and predicted binding affinity (Sethi et al., 2026). The analysis revealed distinct residue-specific energetic contributions, with selected residues showing favourable (negative) energy contributions to ligand binding. These results provide additional energetic insight into the residues potentially involved in stabilising the respective protein–ligand complexes and complement the interaction and MD analyses (**Figure 8**).

**Figure 6:**
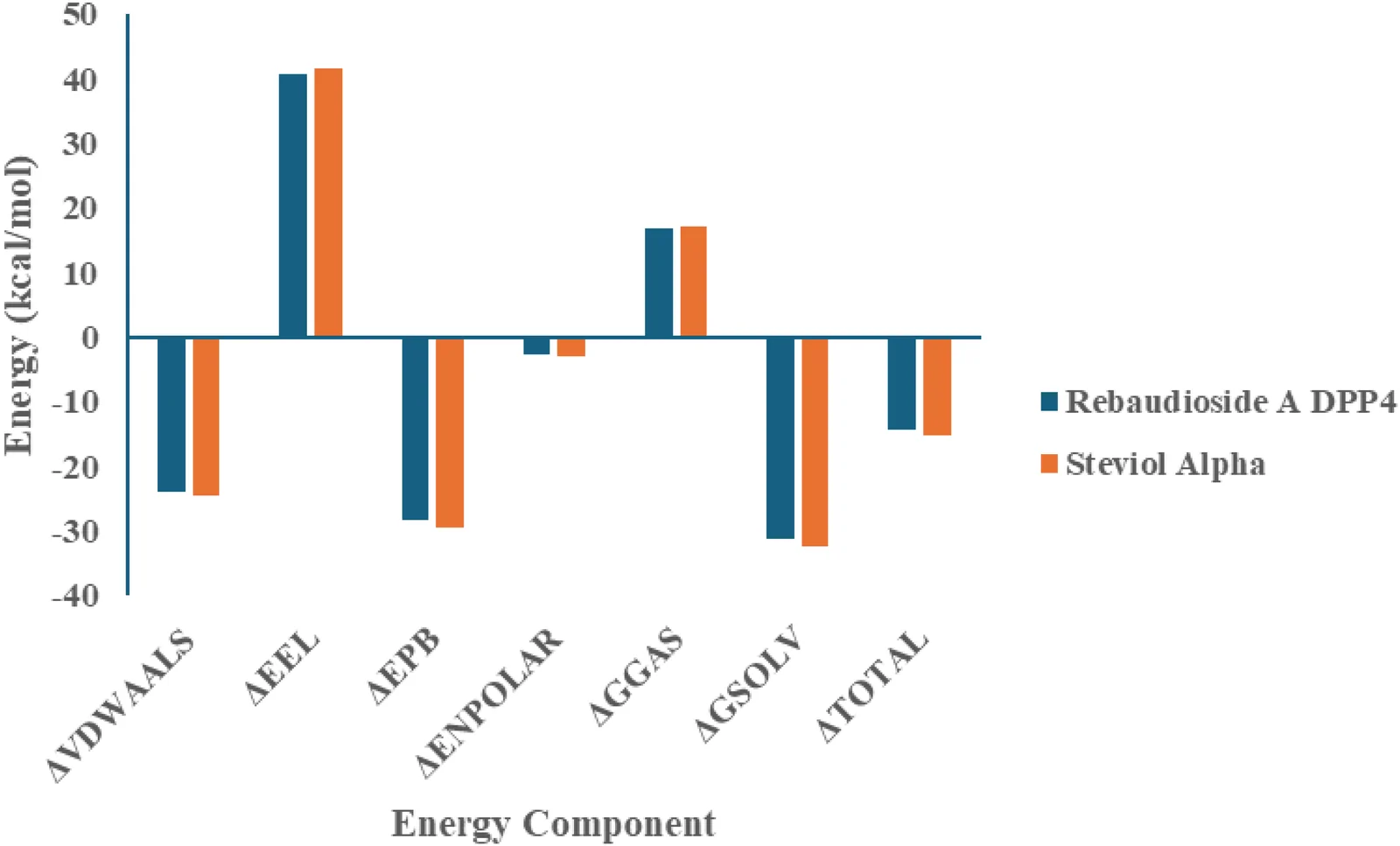
MMPBSA graph for interactions in steviol and Rebaudioside A.

**Figure 7:**
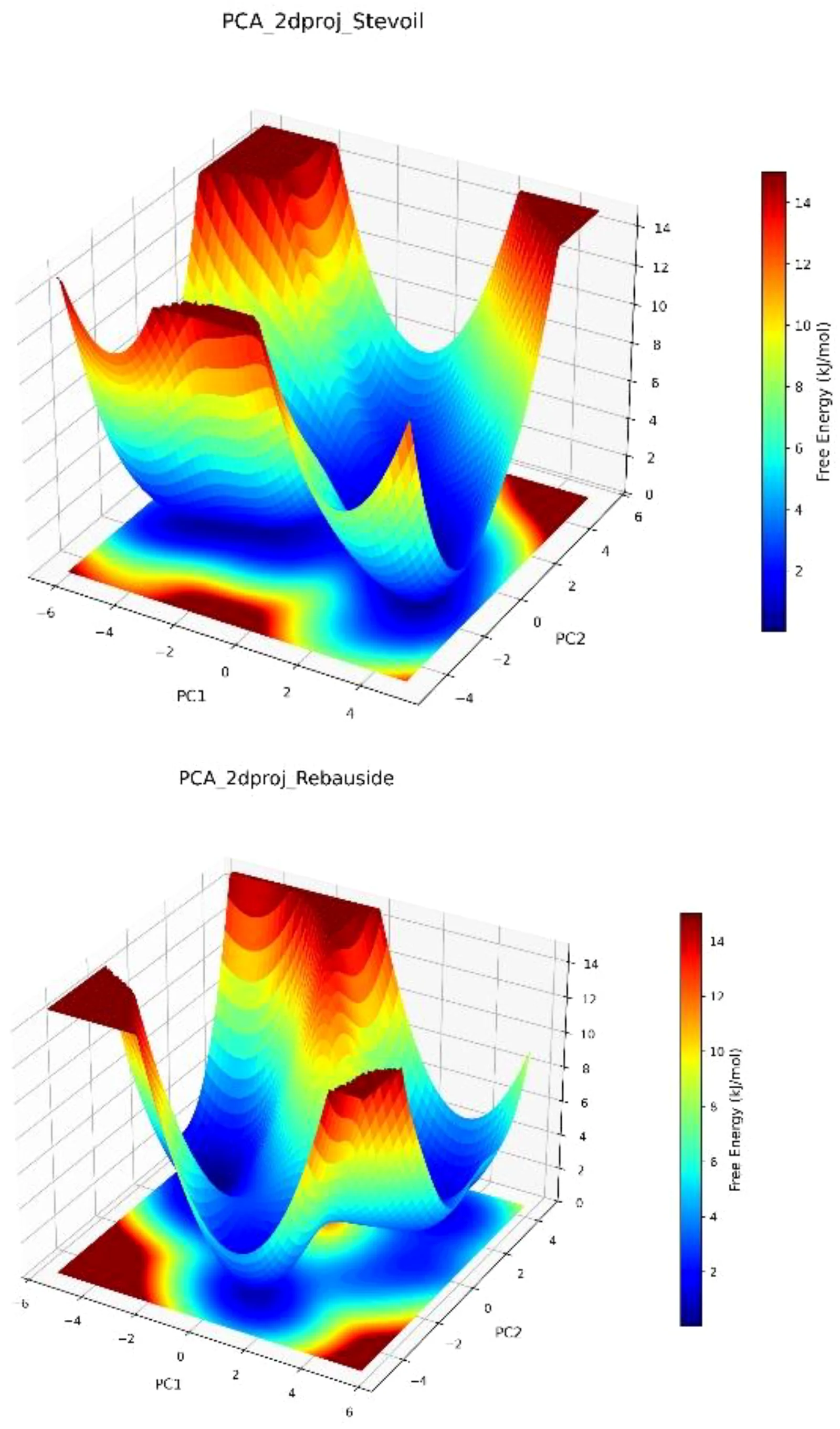
Principal component analysis (PCA) and free-energy landscape of steviol and rebaudioside A protein–ligand complexes.

**Figure 8:**
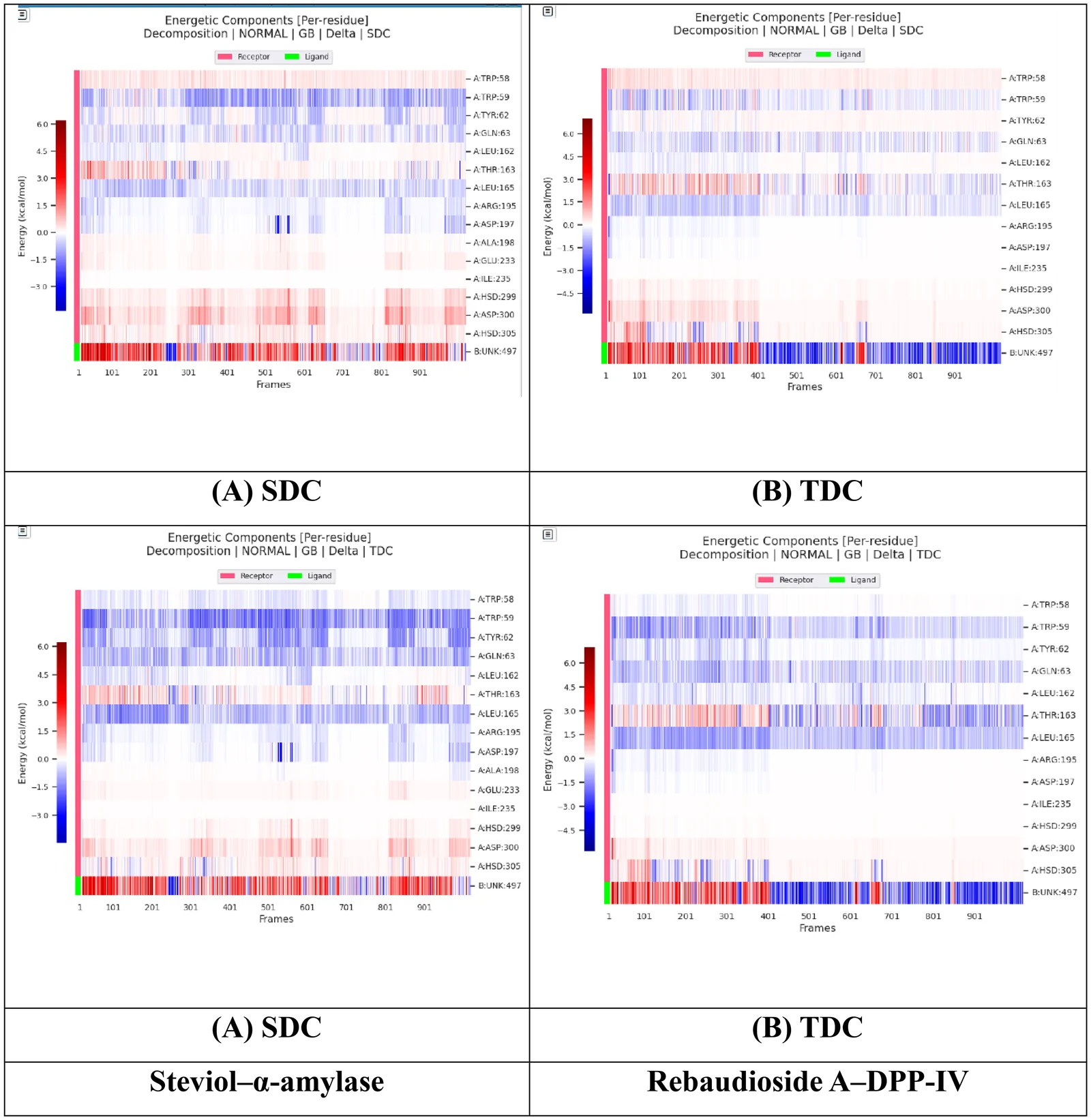
Residue-wise energy decomposition of the steviol–α-amylase and Rebaudioside A–DPP-IV complex **(A)** SDC; **(B)** TDC.

#### 3.5.6 Hydrogen-bond analysis during MD simulation

Hydrogen-bond analysis during the 100 ns MD simulation showed fluctuating and predominantly transient interactions between steviol and α-amylase. The highest occupancies were observed for interactions involving His101 and Thr163, although their relatively low occupancies indicated that these interactions were intermittent rather than persistent. Although no conventional hydrogen bonds were identified for steviol in the initial docking pose, hydrogen-bond analysis during the 100 ns MD simulation revealed transient interactions with several protein residues. The highest occupancies were observed for interactions involving HSD101 (5.09%) and THR163 (4.70%), while the remaining hydrogen-bond interactions showed relatively low occupancies (≤1.70%). Overall, the MD results suggest that hydrogen bonding contributed transiently during the trajectory rather than representing the predominant interaction mode of the complex (**Figure 9**).

**Figure 9:**
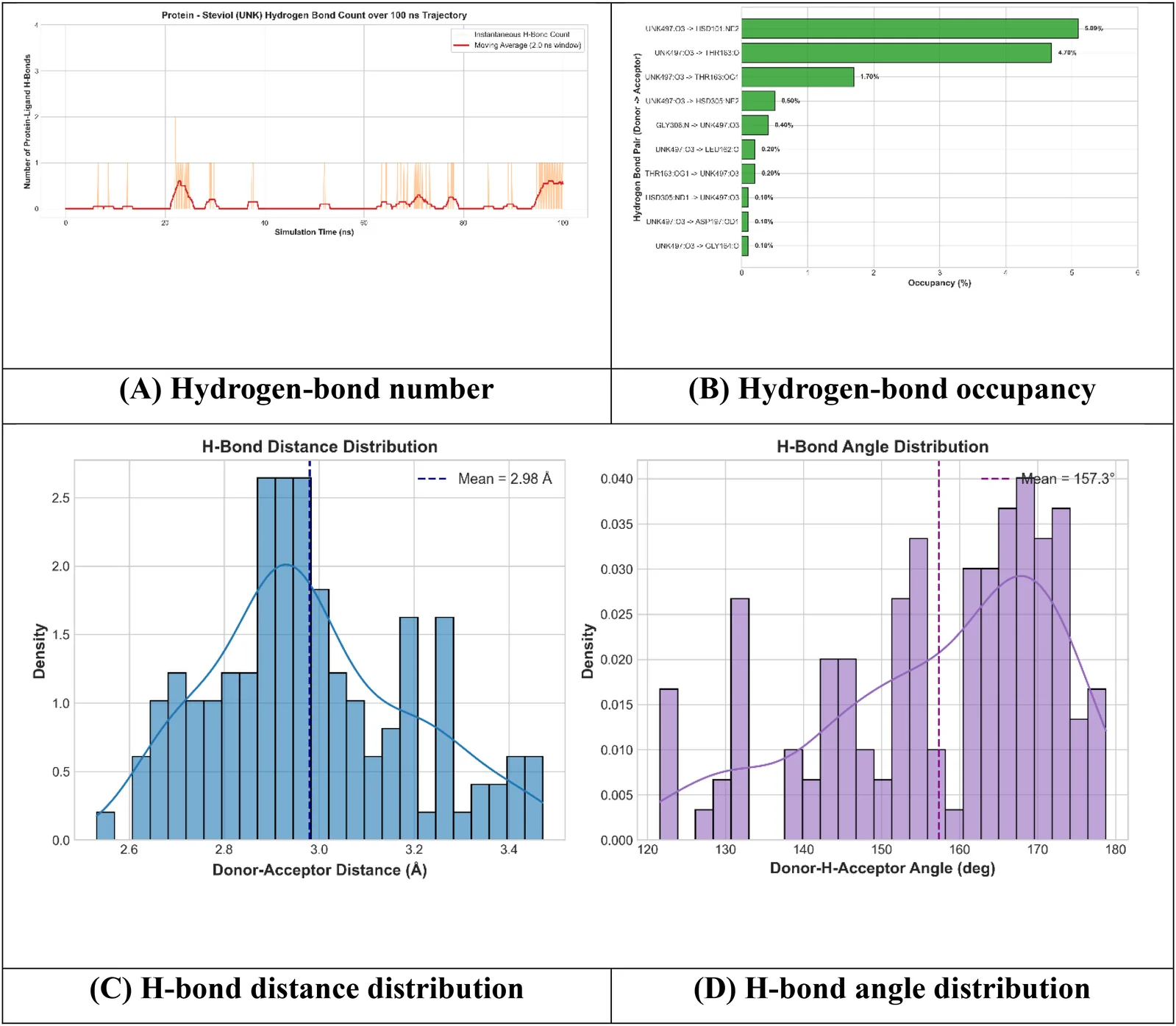
Hydrogen-bond analysis during the 100 ns MD simulation. **(A)** Hydrogen-bond number, **(B)** hydrogen-bond occupancy, **(C)** distance distribution, and **(D)** angle distribution.

#### 3.5.7 MM-PBSA

The MM-PBSA binding free energy analysis indicated favourable predicted binding for both protein–ligand complexes, with the calculated binding energies predominantly influenced by van der Waals and solvation contributions (**Figure 6**). For the steviol–α-amylase complex, favourable van der Waals interactions (ΔEVDWAALS ≈ −24 kcal/mol) and non-polar solvation energy (ΔENPOLAR ≈ −3 kcal/mol) contributed to complex formation, whereas the electrostatic term (ΔEEL ≈ +41 kcal/mol) contributed unfavourably to the calculated binding free energy. This unfavourable electrostatic contribution was partially offset by the favourable polar solvation energy (ΔEPB ≈ −28 kcal/mol), resulting in an overall predicted binding free energy (ΔGTOTAL) of −14.36 ± 3.37 kcal/mol. These results indicate that the predicted interaction between steviol and α-amylase is primarily stabilised through van der Waals and solvation effects rather than electrostatic interactions. The MM-PBSA binding free energy analysis indicated a favourable predicted interaction between rebaudioside A and DPP-IV (**Figure 6**). The calculated binding free energy was primarily influenced by favourable van der Waals interactions (ΔEVDWAALS = −24.52 kcal/mol) together with favourable polar (ΔEPB = −29.52 kcal/mol) and non-polar solvation contributions (ΔENPOLAR = −2.86 kcal/mol). In contrast, the electrostatic component (ΔEEL = +41.73 kcal/mol) contributed unfavourably to the calculated binding free energy. The combined contributions resulted in an overall predicted binding free energy (ΔGTOTAL) of −15.17 ± 3.31 kcal/mol (SD), suggesting a favourable predicted interaction between rebaudioside A and DPP-IV. The negative binding free energy is consistent with a favourable predicted interaction and is broadly consistent with the molecular docking and molecular dynamics analyses. However, it should be noted that MM-PBSA provides an end-state approximation of binding free energy and does not explicitly account for configurational entropy and should therefore be interpreted as an approximate estimate of relative binding free energy rather than an absolute measure of binding affinity. Furthermore, the analysis was performed on a single 100 ns production trajectory; therefore, the reported binding free energy should be interpreted as an approximate computational estimate rather than a definitive measure of binding affinity, and additional independent simulation replicates would be required to assess the reproducibility of these observations. Per-residue energy decomposition identified favourable energetic contributions from residues within the predicted binding sites, broadly consistent with the docking and LigPlot+ interactions. These results provide additional insight into ligand recognition under the simulated conditions.

#### 3.5.8 Dynamic cross-correlation analysis

Dynamic cross-correlation analysis revealed regions of positively and negatively correlated motions within the protein–ligand complex, indicating coordinated and oppositely directed residue movements during the 100 ns MD simulation. The DCCM showed heterogeneous patterns of positive and negative correlations across different regions of the protein, suggesting that ligand-bound dynamics involve both coordinated and oppositely directed residue motions. These correlated motions provide additional information on the dynamic behaviour of the complex and complement the RMSD, RMSF and PCA analyses (**Figure 10**).

**Figure 10:**
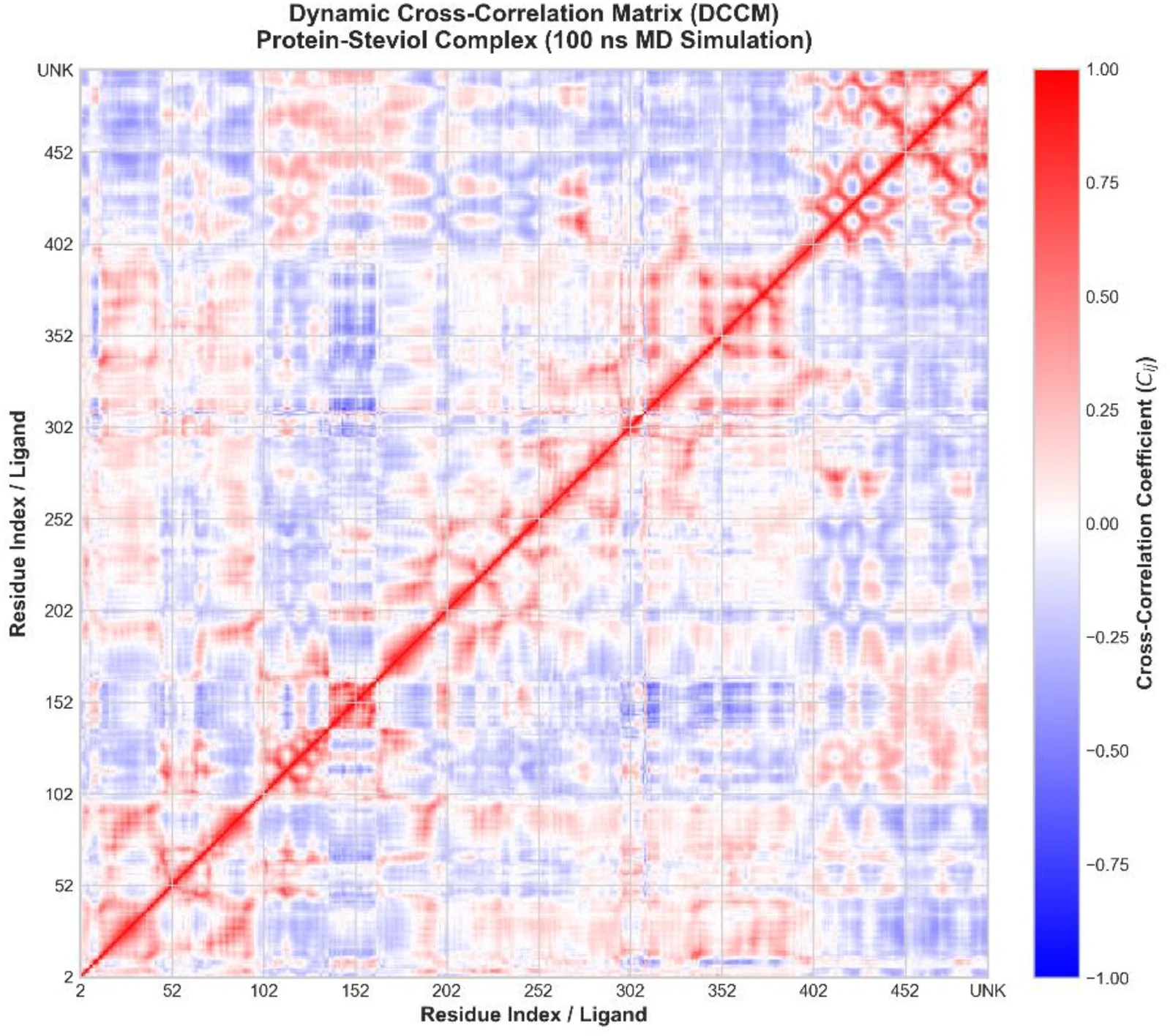
Dynamic cross-correlation matrix (DCCM) of protein Cα atoms during the 100 ns MD simulation of the steviol–protein complex. Red and blue regions represent positively and negatively correlated motions, respectively, while values near zero indicate weak correlation.

### 3.6 Mechanism of action of α-amylase and DPP IV

Natural inhibitors of carbohydrate-digesting enzymes may help limit dietary carbohydrate absorption with fewer adverse effects than synthetic drugs. In particular, pancreatic α-amylase inhibitors derived from natural plant extracts represent a potential approach for diabetes management (Etxeberria et al., 2012). α-Amylase plays a crucial role in polysaccharide hydrolysis and is considered a key digestive enzyme, primarily found in saliva and pancreatic secretions (Mechchate et al., 2021). Inhibition of α-amylase is an established strategy for reducing postprandial hyperglycaemia by delaying carbohydrate digestion (Alqahtani et al., 2020). Pancreatic blocking of α-amylase is a treatment method to delay the breakdown of oligosaccharides to digestible monosaccharides in the brush border of the intestines, reducing postprandial hyperglycaemia (Oyedemi et al., 2017). α-Amylase catalyses the hydrolysis of dietary starch into maltose and other oligosaccharides. By blocking this enzyme, the breakdown of complex carbohydrates into absorbable sugars is slowed down, resulting in a reduced and slower increase in blood glucose levels after eating. α-amylase, in addition to its enzymatic activity, has been known to bind to N-glycans of the duodenal brush-border membrane, which affects glucose uptake. This binding has the potential to suppress sodium-glucose cotransporter 1 (SGLT1) at high concentrations, decreasing glucose absorption in the small intestine (Date et al., 2015; Pierzynowski et al., 2023). Increased levels of α-amylase in the blood have been linked to the inhibition of the secretion of insulin (Akshatha et al., 2021; Chhabria et al., 2022; Ogunyemi et al., 2022). This mechanism could be used to preserve the function of pancreatic beta cells, which would help to postpone the onset of type 2 diabetes (Pierzynowski et al., 2023).

DPP-4 rapidly degrades incretin hormones, primarily glucagon-like peptide-1 (GLP-1) and glucose-dependent insulinotropic polypeptide (GIP), which the gut releases after eating. These hormones have a glucose-dependent stimulatory effect on insulin secretion and an inhibitory effect on glucagon release. The degradation of GLP-1 and GIP is inhibited, thus causing the prolonged activity of both hormones and improved glycaemic control (Thornberry and Gallwitz, 2009). The glucose-dependent stimulation of insulin secretion by pancreatic beta cells due to the increased levels of active GLP-1 and GIP reduce the risk of hypoglycemia, compared to some other antidiabetic agents (Vella, 2012). The high level of incretin also inhibits glucagon secretion by alpha cells, that decrease hepatic level of glucose, which helps in reducing the level of blood glucose (Gallwitz, 2019).

## 4 Conclusion and future prospects

This study employed an integrated *in silico* workflow comprising molecular docking, molecular dynamics simulations, PCA, free-energy landscape analysis, DCCM, hydrogen-bond analysis, MM-PBSA, per-residue energy decomposition, and ADME analyses to evaluate the predicted interactions of selected steviol-derived phytochemicals with human pancreatic α-amylase and DPP-IV. Among the evaluated phytochemicals, steviol and rebaudioside A exhibited the most favourable predicted docking profiles against α-amylase and DPP-IV (binding energies of −8.46 and −8.34 kcal/mol, respectively). Molecular dynamics and complementary structural and energetic analyses provided additional insights into the conformational behaviour, intermolecular interactions, correlated motions, and residue-level energetic contributions of the selected complexes. Despite its favourable predicted binding affinity toward DPP-IV (−8.34 kcal/mol), rebaudioside A exhibited comparatively less favourable predicted pharmacokinetic properties, including a low predicted bioavailability score (0.11) and multiple Lipinski’s rule violations. These findings suggest that, although rebaudioside A demonstrates promising target affinity *in silico*, additional pharmacokinetic evaluation would be required to assess its suitability as an orally administered therapeutic agent. Accordingly, the distinction between target affinity and predicted drug-likeness should be considered when interpreting its therapeutic potential. The present computational findings are consistent with previous experimental studies demonstrating that steviol glycosides improve glycaemic control, insulin sensitivity, and glucose homeostasis in experimental models of diabetes (Anker et al., 2019). In addition, previous molecular docking studies have also reported favourable interactions of steviol-derived compounds with key antidiabetic targets, supporting the biological relevance of the present findings. The present findings are derived entirely from computational analyses and therefore represent predicted molecular interactions rather than experimentally validated enzyme inhibition or therapeutic efficacy. Overall, the present study identifies steviol and rebaudioside A as promising lead phytochemicals for further antidiabetic investigation while emphasizing that these findings remain computational predictions requiring experimental validation. Future studies should validate these computational findings through *in vitro* α-amylase and DPP-IV inhibition assays, followed by structure–activity relationship studies, experimental pharmacokinetic evaluation, and *in vivo* investigations to establish their biological and translational relevance. 

**Table 7:** Hydrogen bond interactions of the DPP IV and identified phytocompounds complex.

S. No	Pub ID	Ligand x 3OLD	Binding affinity (kcal/mol)	No. of H bonds	Amino acids involved in H bonding
1	6918840	Rebaudioside A	-8.34	7	GLU 361, ARG 358, TYR 585, GLU 206, TYR 662, TYR 547, SER 630
2	71773169	Rebaudioside D	-5.95	5	PHE 208, TRP 215, SER 212. ILE 107, TRP 62
3	452967	Steviol (Hydroxydehydrostevic acid)	-4.42	–	–
4	442089	Stevioside	-6.33	4	SER 212, GLU 261, SER 106, ILE 107

## Data Availability

The data presented in the study are deposited in the Figshare repository, DOI: https://doi.org/10.6084/m9.figshare.33870481.
